# Renal function and outcomes in atrial fibrillation patients after catheter ablation

**DOI:** 10.1371/journal.pone.0241449

**Published:** 2020-11-09

**Authors:** Tetsuma Kawaji, Satoshi Shizuta, Takanori Aizawa, Shintaro Yamagami, Yasuaki Takeji, Yusuke Yoshikawa, Masashi Kato, Takafumi Yokomatsu, Shinji Miki, Koh Ono, Takeshi Kimura

**Affiliations:** 1 Department of Cardiology, Ryorei Memorial Kyoto Hospital, Kyoto, Japan; 2 Department of Cardiovascular Medicine, Graduate School of Medicine, Kyoto University, Kyoto, Japan; International University of Health and Welfare, School of Medicine, JAPAN

## Abstract

**Background:**

Atrial fibrillation (AF) and renal failure coexist and interact. However, scarce data about association between renal function and clinical outcomes in patients undergoing catheter ablation for AF are available. We sought to evaluate long-term renal function and clinical outcomes after AF ablation.

**Methods:**

We enrolled 791 non-dialysis patients undergoing catheter ablation for AF, and evaluated the incidence of worsening renal function (WRF) after the procedure, defined as >30% decline in estimate glomerular filtration rate.

**Results:**

Mean follow-up duration was 5.1±2.5 years. Five hundreds and twenty-six patients (66.5%) were free from recurrent atrial arrhythmias without any antiarrhythmic drugs at the time of final follow-up. Cumulative incidence of WRF was 13.2% at 5-year after procedure, which was significantly higher in patients with recurrent AF compared to those without (21.6% versus 8.7%, P<0.001). In the multivariable analysis, recurrent AF was an independent risk factor for WRF (adjusted hazard ratio [HR] 1.89, 95% confidence interval 1.27–2.81, P = 0.002), along with congestive heart failure, diabetes, and eGFR <60 ml/min/1.73m^2^ at baseline. Patients with WRF had significantly higher 5-year incidences of all-cause death, cardiovascular death, heart failure hospitalization, ischemic stroke, and major bleeding compared to those without WRF. After adjustment of baseline differences in the multivariate Cox model, the excessive risks of WRF for all-cause death and heart failure hospitalization remained significant (adjusted HR 3.46, P = 0.002; adjusted HR 3.67, P<0.001).

**Conclusions:**

In AF patients undergoing catheter ablation for AF, arrhythmia recurrence was associated with WRF during follow-up, which was a strong predictor of adverse clinical outcomes.

## Introduction

Atrial fibrillation (AF) is the most common arrhythmia in daily clinical practice. The prevalence of AF is well known to increase with age. Other known risk factors for development of AF includes hypertension, diabetes, and cardiovascular diseases, which have been also identified as risk factors for chronic kidney disease (CKD) [1–3]. Also, existence of AF increases the risk of development of CKD, and conversely, CKD increases the risk of new onset AF [4]. Thus, there is a significant bidirectional association between AF and CKD.

Catheter ablation, as well as surgical MAZE procedure, could eliminate AF and may break the vicious cycle between AF and CKD. Takahashi et al. reported that elimination of AF by catheter ablation improved renal function over a 1-year follow-up period in AF patients with CKD [5]. Park et al., furthermore, reported that AF ablation improved 5-year renal function compared with medical therapy [6]. In addition, Kornej et al. reported that eGFR change after AF ablation was associated with baseline CHA_2_DS_2_-VASc score and AF recurrences during 20 months of follow-up [7]. However, the association between long-term renal function and clinical outcomes after ablation for AF has not been fully evaluated. We, therefore, sought to elucidate the association between recurrent AF and worsening renal function (WRF) during long-term follow-up after catheter ablation for AF from a large single-center database [8], especially focusing on the impact of WRF on clinical outcomes.

## Methods

### Study design

Among 1206 consecutive patients undergoing first radiofrequency catheter ablation for AF in Kyoto University Hospital between February 2004 and March 2015, we excluded 21 patients receiving hemodialysis, 1 patient whose serum creatinine (SCr) value at the time of procedure was not available, 7 patients who died within 6 months post procedure, and 386 patients whose SCr value beyond 6 months post ablation were not available. Accordingly, we enrolled 791 non-dialysis patients with AF to validate long-term renal function after catheter ablation in the present study.

### Ethics

Written informed consent for the ablation procedure and follow-up was obtained from all patients. Follow-up information was obtained by review of hospital-chart and/or telephone contact with the patient, relatives, and/or referring practitioners. The study protocol was approved by the institutional review board of Kyoto University Hospital.

### Ablation and follow-up protocol

We have previously reported the detailed protocol of catheter ablation procedure in the study population [8]. In brief, antiarrhythmic drugs (AADs) were discontinued at least >24 hours before the procedure. Extensive encircling pulmonary veins isolation was performed. Tricuspid valve isthmus ablation was routinely performed regardless of the presence of typical atrial flutter. Superior vena cava isolation and substrate modification were added whenever necessary. Additional complex fractionated atrial electrogram ablation was performed when sinus restoration was not obtained after pulmonary veins isolation or AF was easily induced by electrical stimulation in the atrium and/or isoproterenol infusion. Additional left atrial linear ablations were performed for sustained atrial tachycardias during the procedure.

A 12-lead electrocardiogram was routinely measured at each clinical visit, and 24-hour Holter monitoring was recommended at 3-, 6-, 12-month and at least annually thereafter. Additional 24-hour Holter monitoring and/or ambulatory electrocardiogram were recorded when patients had symptoms. SCr measurement was recommended every 3 months during the first year after ablation and at least annually thereafter.

Oral anticoagulant (OAC) was recommended to have been administered more than 1 month before ablation and to be continued for at least 3 months after the procedure. Thereafter, discontinuation of OAC in patients without arrhythmia recurrence was left to the discretion of the attending physician. Also, whether to administer AADs after procedure was left to the discretion of the attending physician. When recurrent atrial tachyarrhythmias were detected after the blanking period of 3 months post ablation, the repeat procedures were recommended to the patients.

### Definitions and outcome measures

Because study patients were all Japanese in the present study, estimate glomerular filtration rate (eGFR) was calculated by the Japanese Society of Nephrology-Chronic Kidney Disease Initiatives (JSN-CKDI) equation, which is the official eGFR formula in Japan [9].

eGFR [ml/min/1.73m^2^] = 194 × SCr^-1.094^ × Age^-0.287^ × 0.739 (if female)

Baseline CKD was defined as eGFR <60 ml/min/1.73m^2^ at the time of the first ablation procedure. The primary outcome measure was WRF defined as >30% decline in eGFR at any time during the follow-up period after the first procedure, according to the recommendation of the National Kidney Foundation and the Food and Drug Administration in 2012, i.e. 30–40% decline in eGFR over 2–3 years follow-up (10–15% decline per year) [10, 11]. Furthermore, in the sensitivity analysis of annual eGFR decline, WRF was defined as >10% annual decline in eGFR during follow-up period.

The type of AF was classified into paroxysmal (lasting <7 days) and non-paroxysmal (lasting ≥7 days). Recurrent AF was defined as the presence of recurrent atrial tachyarrhythmias at the time of last follow-up. The recurrent atrial tachyarrhythmias were defined as documented atrial tachyarrhythmias lasting for >30 seconds or those requiring repeat ablation procedures with a blanking period of 90 days post ablation procedure. Maintained sinus rhythm was defined as free from recurrent atrial tachyarrhythmias without any AADs during follow-up duration. AADs only included Vaughan Williams class I or III drugs. Sinus rhythm maintained under any AADs was regarded as recurrent AF. Discontinuation of OAC was regarded as present when it was intended to be permanent. Baseline congestive heart failure (CHF) was defined as hospitalization for exacerbation of HF before the index ablation procedure and/or left ventricular ejection fraction of <40%. The secondary clinical outcome measures were all-cause death, cardiovascular death, heart failure hospitalization, ischemic stroke, and major bleeding. Death was regarded as cardiac in origin unless obvious non-cardiac causes could be identified. Ischemic and hemorrhagic strokes were distinguished by imaging studies. Major bleeding was defined as International Society of Thrombosis and Hemostasis (ISTH) major bleeding [12].

### Statistical analysis

Categorical variables were presented as number and percentage and were compared with the chi-square test when appropriate; otherwise, we used Fisher’s exact test. Continuous variables were presented as mean and standard deviation or median with interquartile range, and were compared using the Student’s t-test or Wilcoxon rank sum test based on their distributions. We used the Kaplan-Meier method to estimate 5-year cumulative incidence, and assessed the difference with the log-rank test. Multivariable analyses using the Cox proportional hazard model with 11 clinically relevant variables (WRF, recurrent AF, age >75 year old, body mass index >25 kg/m^2^, non-paroxysmal AF, female, hypertension, diabetes, CHF, baseline eGFR <60 ml/min/1.73m^2^, and Warfarin use) were conducted to identify independent risk factors for WRF and all clinical outcomes following the procedure. Because of the limited number of events, only variables with P<0.05 on univariable analysis were included. To account for competing risk of all-cause death, we constructed Fine-Gray subdistribution hazard models [13, 14] with the same covariates in the main analysis as a sensitivity analysis. Continuous variables were dichotomized by clinically meaningful reference values. Statistical analyses were performed using JMP 10 (SAS Institute Inc, Cary, NC) and R version 3.6.1. (R Foundation for Statistical Computing, Vienna, Austria). All analyses were two-tailed, and P value of <0.05 was considered statistically significant.

## Results

### Patient characteristics

Mean age of the present study population was 64.6±9.6 years old (Table 1). The prevalence of paroxysmal AF and CHF were 69.4% and 9.9%, respectively. Mean eGFR was 63.0±12.4 ml/min/1.73m^2^ and the prevalence of CKD was 32.3% at baseline. Most patients (95.3%) were administered OAC at discharge, and about half of OAC was warfarin.

**Table 1:** Patient characteristics.

	Overall, N = 791	Maintained sinus rhythm, N = 526 (66.5%)	Recurrent atrial fibrillation, N = 265 (33.5%)	P value
Age (years)	64.6±9.6	64.0±9.6	65.6±9.5	0.03
Body mass index (kg/m^2^)	23.8±3.6	23.7±3.4	24.1±3.9	0.09
AF duration (years)	2.4 [0.7–6.0]	2.1 [0.6–5.4]	3.0 [1.0–7.5]	<0.001
Paroxysmal AF	549 (69.4%)	399 (75.9%)	150 (56.6%)	<0.001
Female	246 (31.1%)	147 (28.0%)	99 (37.4%)	0.007
Hypertension	459 (58.0%)	298 (56.7%)	161 (60.8%)	0.27
Diabetes	127 (16.1%)	80 (15.2%)	47 (17.7%)	0.36
Ischemic stroke	79 (10.0%)	46 (8.8%)	33 (12.5%)	0.11
Congestive heart failure	78 (9.9%)	37 (7.2%)	40 (15.1%)	<0.001
eGFR (ml/min/1.73m^2^)	67.8±16.9	68.6±17.2	66.2±16.2	0.053
Baseline CKD (eGFR< 60 ml/min/1.73m^2^)	255 (32.3%)	160 (30.4%)	95 (35.9%)	0.12
CHADS_2_ score	1.2±1.1	1.1±1.0	1.4±1.1	<0.001
CHA_2_DS_2_-VASc score	2.1±1.5	1.9±1.5	2.4±1.6	<0.001
**Echocardiography**				
Left ventricular ejection fraction (%)	63.0±12.4	64.1±11.1	60.9±14.5	<0.001
Left atrial diameter (mm)	41.1±7.0	40.1±6.6	43.2±7.5	<0.001
**Medications at discharge**				
Oral anticoagulation	754 (95.3%)	504 (95.8%)	250 (94.3%)	0.36
Warfarin	415 (52.5%)	242 (46.0%)	173 (65.3%)	<0.001
DOACs	339 (42.9%)	262 (49.8%)	77 (29.1%)	<0.001
Antiplatelets	163 (20.6%)	90 (17.1%)	73 (27.6%)	<0.001
ACE-I/ARB	334 (42.2%)	211 (40.1%)	123 (46.4%)	0.09
Beta blockers	268 (33.9%)	172 (32.7%)	96 (36.2%)	0.32

ACE-I = angiotensin converting enzyme inhibitor; AF = atrial fibrillation; ARB = angiotensin receptor blocker; CKD = chronic kidney disease; DOACs = direct oral anticoagulants; eGFR = estimated glomerular filtration rate.

Mean follow-up duration was 5.1±2.5 years. During the follow-up period, repeat procedures were performed in 347 patients (43.9%) (S1 Fig). Vast majority of the second procedures (73.5%) were performed within 1.5 years after the first procedure. At the final follow-up, 526 patients (66.5%) were free from recurrent AF without AADs. The cumulative incidence of OAC discontinuation at 5-year was 55.8%.

Patients with recurrent AF had higher prevalence of elderly (≥75 years old), non-paroxysmal AF, female, and CHF compared with maintained sinus rhythm patients, meanwhile the prevalence of baseline CKD was not significantly different between the 2 groups. The prevalence of warfarin and antiplatelets use at the time of discharge was significantly higher in patients with recurrent AF.

### Worsening renal function after ablation

**Fig 1:**
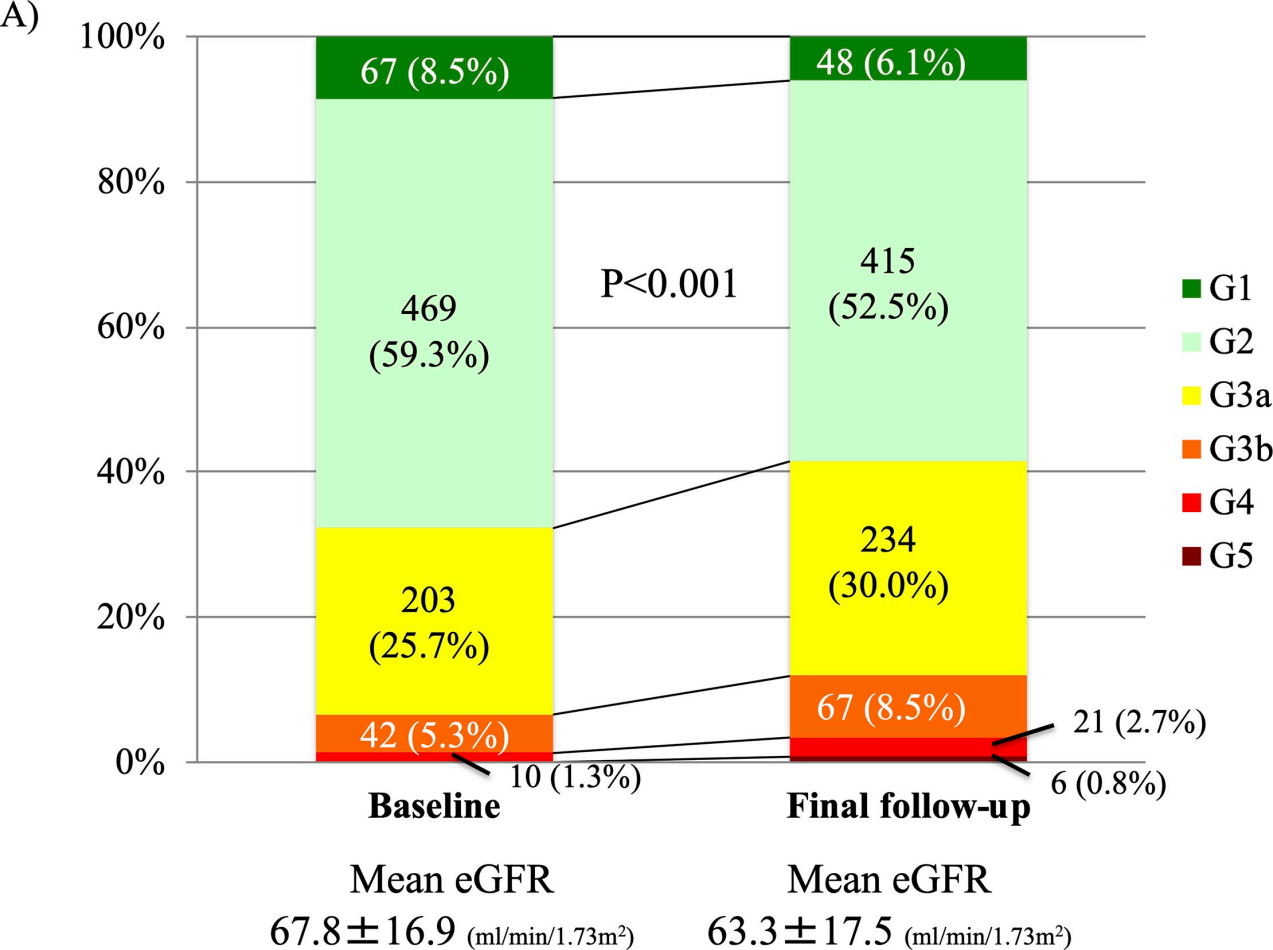
Changes in chronic kidney disease stage during follow-up among A) overall study population, and B) patients with and without recurrent AF.

**Fig 2:**
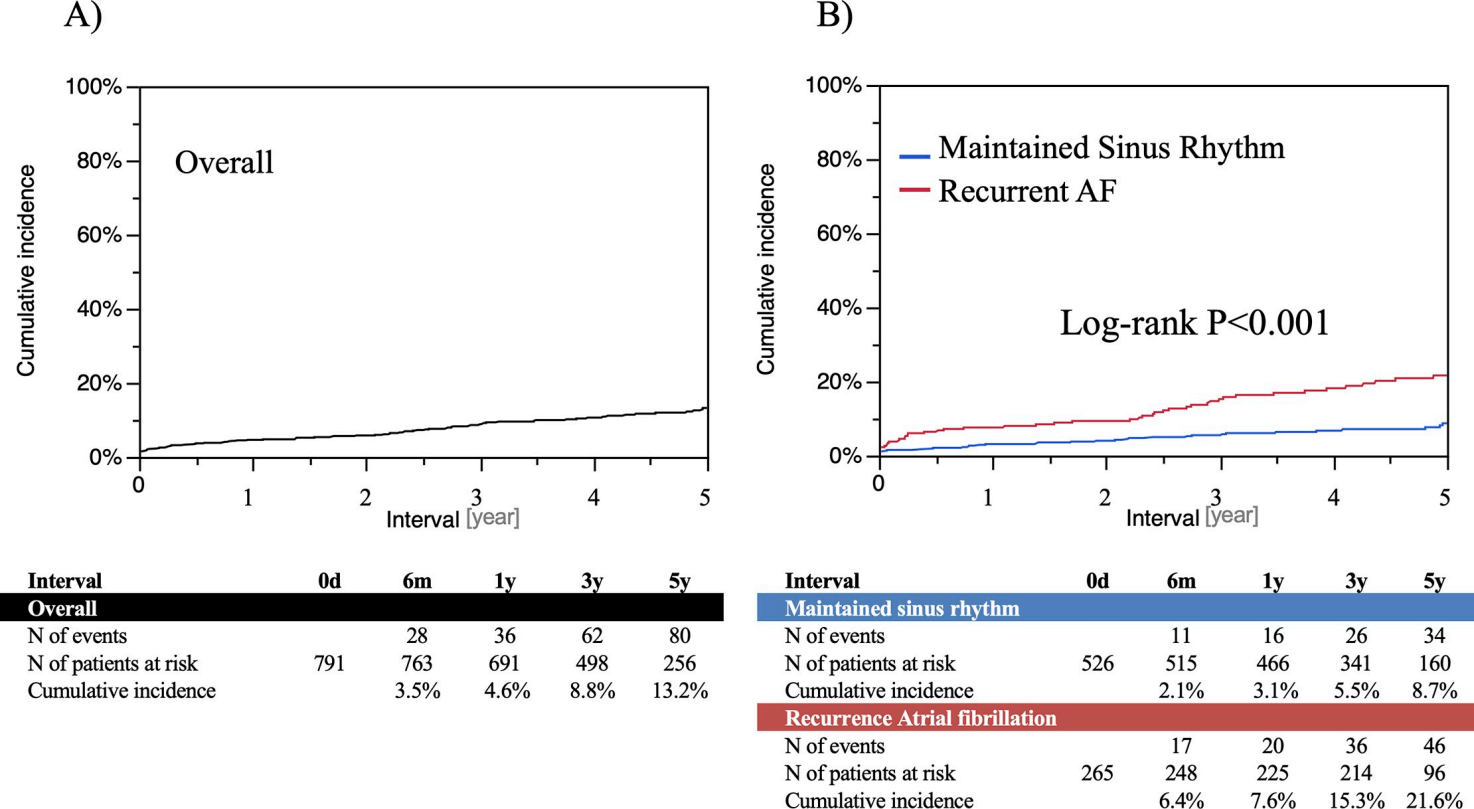
Cumulative incidence of worsening renal function after AF ablation among A) overall study population, and B) patients with and without recurrent AF.

eGFR decreased from 67.8±16.9 ml/min/1.73m^2^ at baseline to 63.3±17.5 ml/min/1.73m^2^ at final follow-up (P<0.001), and the prevalence of CKD ≥stage 3 increased from 31.0% to 38.5% (Fig 1). The prevalence of CKD stage 3b at final follow-up was higher in patients with recurrent AF compared to those with maintained sinus rhythm (17.4% vs. 4.0%, P<0.001). The cumulative incidences of >10%, >20%, and >30% declines in eGFR after catheter ablation for AF were shown in S2 Fig. The cumulative incidence of WRF defined as >30% decline in eGFR was 3.5%, 8.5%, and 13.2% at 1-, 3-, and 5-year, respectively (Fig 2). The 5-year incidence of WRF in patients with recurrent AF was significantly higher than in those with maintained sinus rhythm (21.6% versus 8.7%, P<0.001). The independent risk factors for WRF after procedure included recurrent AF (hazard ratio [HR] 1.89, 95% confidence interval [CI] 1.27–2.81, P = 0.002), diabetes (HR 1.83, 95%CI 1.18–2.83, P = 0.01), CHF (HR 3.00, 95%CI 1.92–4.69, P<0.001), and baseline CKD (HR 1.52, 95%CI 1.01–2.27, P = 0.046) (Table 2). In the both sensitivity analyses with all-cause death as a competing risk and excluding AF patients with baseline CKD, recurrent AF as well as diabetes and CHF became independent predictors for WRF (adjusted HR 3.00, 95%CI 1.89–2.82, P = 0.002; adjusted HR 2.10, 95%CI 1.23–3.61, P = 0.007) (S1 and S2 Tables).

**Table 2:** Independent risk factors for WRF after catheter ablation for AF.

Variables	HR	95% CI	P value
**Recurrent AF**	1.89	1.27–2.81	0.002
**Age >75 years old**	1.10	0.66–1.85	0.71
**Body mass index >25 kg/m**^**2**^	0.89	0.58–1.36	0.59
**Non-paroxysmal AF**	1.23	0.81–1.86	0.33
**Female**	1.22	0.82–1.83	0.33
**Hypertension**	1.45	0.96–2.20	0.07
**Diabetes**	1.83	1.18–2.83	0.01
**Congestive heart failure**	3.00	1.92–4.69	<0.001
Baseline CKD*	1.52	1.01–2.27	0.046
**Warfarin use**	1.07	0.70–1.63	0.77

CI = confidence interval; HR = hazard ratio; WRF = worsening renal function.

Other abbreviations as in Table 1.

### Clinical outcomes following ablation

The cumulative incidence of OAC discontinuation was significantly lower in patients with recurrent AF (28.0% versus 70.4%, P<0.001) and in those with WRF (41.1% versus 58.4%, P<0.001) (S3 Fig).

**Fig 3:**
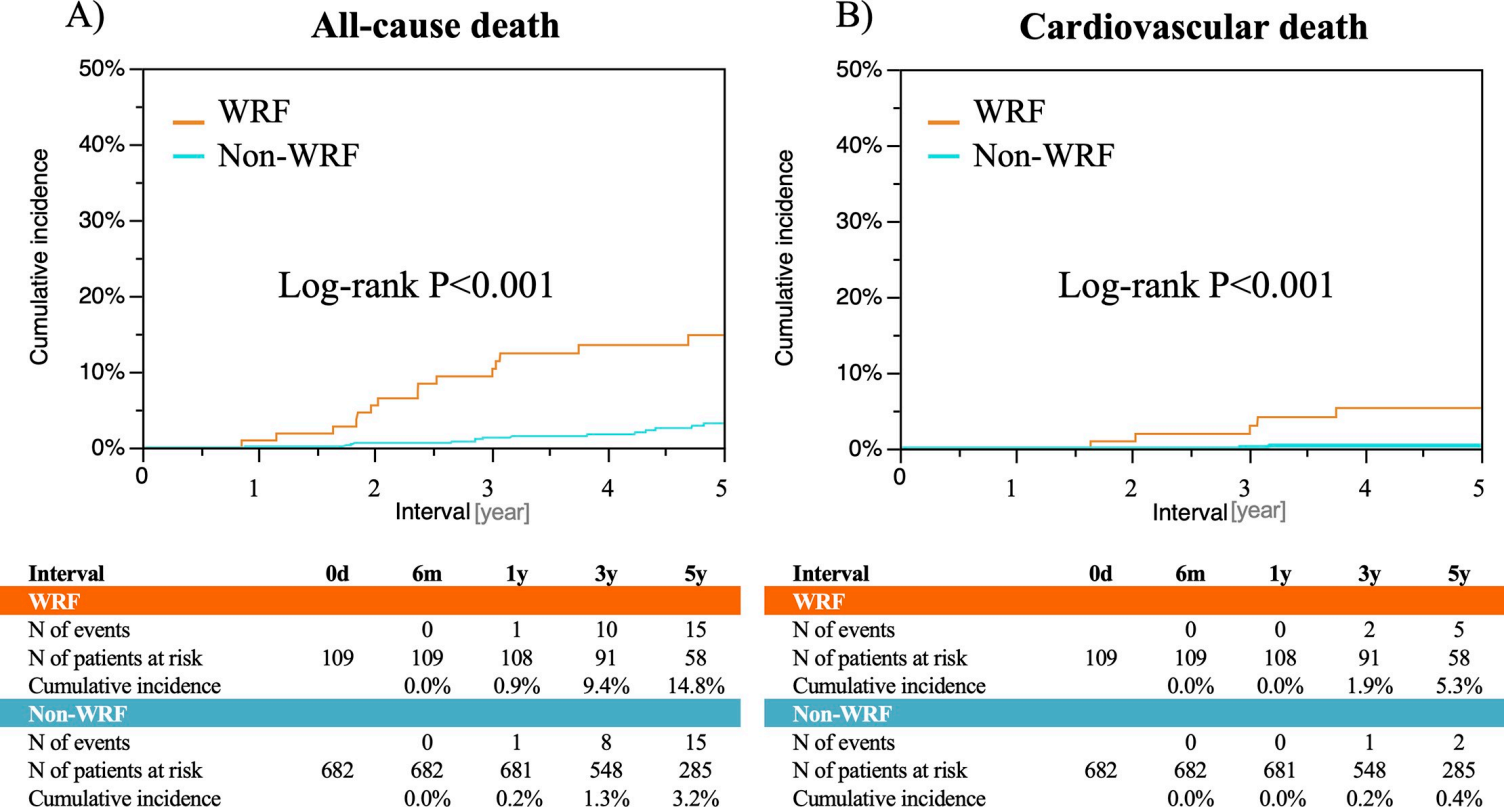
Cumulative incidences of clinical outcomes after catheter ablation for AF comparing patients with and without WRF. A) all-cause death, B) cardiovascular death, C) heart failure hospitalization, D) ischemic stroke, and E) major bleeding. WRF = worsening renal function.

After ablation procedure, patients with WRF had significantly higher 5-year incidence of all-cause death (14.8% versus 3.2%, P<0.001), cardiovascular death (5.3% versus 0.4%, P<0.001), heart failure hospitalization (15.8% versus 2.0%, P<0.001), ischemic stroke (3.7% versus 0.2%, P<0.001), and major bleeding (8.2% versus 0.1%, P<0.001) compared to those without WRF (Fig 3). Furthermore, patients with WRF within 1 year post procedure was associated with significantly higher risk for all-cause death, cardiovascular death, heart failure hospitalization, ischemic stroke, and major bleeding relative to those without (S4 Fig).

In the multivariable analysis, WRF (HR 3.46, 95%CI 1.60–7.36, P = 0.002) as well as baseline CKD (HR 2.12, 95%CI 1.05–4.30, P = 0.04) was an independent predictor for all-cause death (Table 3). Independent risk factors for heart failure hospitalization included WRF (HR 3.67, 95%CI 1.67–8.20, P<0.001), recurrent AF (HR 3.05, 95%CI 1.31–7.96, P = 0.01), age >75 years old (HR 2.85, 95%CI 1.28–6.37, P = 0.02), CHF (HR 6.27, 95%CI 2.94–13.4, P<0.001), and baseline CKD (HR 2.10, 95%CI 1.01–4.38, P = 0.046) (Table 3). In the sensitivity analyses with all-cause death as a competing risk, the results were almost consistent with those of the main analysis except baseline CKD (S3 Table).

**Table 3:** Independent risk factors for all-cause death and heart failure hospitalization after catheter ablation for AF.

Variables	Univariate			Multivariable		
	HR	95% CI	P value	HR	95% CI	P value
**A) All-cause death**						
**WRF**	5.49	2.75–10.9	<0.001	3.46	1.60–7.36	0.002
**Recurrent AF**	2.26	1.08–4.53	0.03	1.38	0.67–2.88	0.39
**Age >75 years old**	1.58	0.59–3.58	0.34			
**Body mass index >25 kg/m**^**2**^	0.81	0.36–1.69	0.59			
**Non-paroxysmal AF**	1.05	0.48–2.14	0.9			
**Female**	0.98	0.45–2.00	0.96			
**Hypertension**	1.03	0.52–2.09	0.94			
**Diabetes**	2.61	1.22–5.27	0.02	1.81	0.83–3.72	0.13
**Congestive heart failure**	4.38	1.98–9.01	<0.001	2.23	0.96–4.89	0.06
Baseline CKD*	2.76	1.39–5.53	0.004	2.12	1.05–4.30	0.04
**Warfarin use**	2.21	1.00–5.59	0.05			
**B) Heart failure hospitalization**						
**WRF**	8.44	4.12–17.7	<0.001	3.67	1.67–8.20	<0.001
**Recurrent AF**	4.73	2.31–9.83	<0.001	3.05	1.31–7.96	0.01
**Age >75 years old**	3.3	1.48–6.89	0.005	2.85	1.28–6.37	0.02
**Body mass index >25 kg/m**^**2**^	1.46	0.68–2.99	0.32			
**Non-paroxysmal AF**	2.36	1.15–4.87	0.02	1.85	0.89–3.87	0.1
**Female**	0.96	0.42–2.04	0.92			
**Hypertension**	1.27	0.62–2.77	0.52			
**Diabetes**	2.32	1.01–4.09	0.048	1.68	0.76–3.72	0.22
**Congestive heart failure**	13.6	6.61–28.5	<0.001	6.27	2.94–13.4	<0.001
Baseline CKD*	3.17	1.54–6.67	0.002	2.1	1.01–4.38	0.046
**Warfarin use**	1.47	0.70–3.31	0.32			

Abbreviations as in Tables 1 and 2.

*Defined as eGFR <60 ml/min/1.73m^2^

### Sensitivity analysis of annual eGFR decline

Median annual rate of eGFR change was -0.6% (-3.5% - 1.7%) and significantly higher in patients with recurrent AF compared to those with maintained sinus rhythm (1.3% vs. 0.5%, P = 0.02) (S5 Fig). The prevalence of WRF defined as >10% annual decline in eGFR was observed in 47 patients (5.9%). Recurrent AF became an independent predictor for WRF (HR 2.19, 95%CI 1.16–4.16, P = 0.02) (S4 Table). Patients with WRF had significantly higher 5-year incidence of all-cause death (19.3% versus 3.9%, P<0.001), cardiovascular death (5.0% versus 0.9%, P<0.001), heart failure hospitalization (17.4% versus 3.1%, P<0.001), and ischemic stroke (4.4% versus 0.4%, P = 0.004) compared to those without WRF (S6 Fig). In the multivariable analysis with and without all-cause death as a competing risk, WRF also became an independent predictor for all-cause death and heart failure hospitalization (S5 Table).

## Discussion

The present study evaluated the impact of arrhythmia recurrence following catheter ablation for AF on the long-term renal function, and also assessed the association between WRF and long-term clinical outcomes after procedure. The main findings of the present study were; (1) the cumulative incidence of WRF defined as >30% decline in eGFR after catheter ablation for AF was 13.2% at 5-year, (2) recurrent AF was an independent risk factor for WRF, (3) patients with WRF, especially within 1 year post procedure, had higher incidence of long-term adverse clinical outcomes, (4) history of CHF was also an independent risk factor for WRF and adverse clinical outcomes.

A significant bidirectional association between AF and CKD has been reported in previous studies [3, 4]. CKD is an independent risk factor for new onset AF and AF worsens renal function. Although warfarin had been commonly used in AF patients until development of direct oral anticoagulants (DOACs), its harmful effects on renal function, so-called warfarin-related nephropathy, has been recently recognized [15]. The main mechanism of the warfarin-related nephropathy is considered calcification of renal arteries induced by inhibition of vitamin K-dependent protein matrix gamma-carboxyglutamic acid (Gla/MGP) [16, 17]. Other possible mechanisms include renal infarction and microbleeds in kidney. Previous studies reported that the severity of decline in renal function with warfarin depended on time in therapeutic range [18, 19]. On the other hand, DOACs do not inhibit Gla/MGP, and may be potentially protective for renal function because they inhibit thrombin or factor Xa, which has been demonstrated to be associated with vascular inflammation [20, 21]. Furthermore, DOACs were associated with significantly lower bleeding risks compared with warfarin. Indeed, several studies reported that DOACs as compared with warfarin were associated with lower risks for significant decline in eGFR [11, 22, 23]. In the present study, however, DOACs were not associated with reduced risk for WRF, presumably because of high incidence of OAC discontinuation during follow-up. Even with the use of DOACs, eGFR gradually declines overtime, especially in AF patients, presumably due to age-related degenerations, micro embolism or bleeding, and hypoperfusion in kidney. Thus, restoration and maintenance of sinus rhythm by catheter ablation for AF may be important to minimize decline of renal function over time.

AF ablation restores and maintains sinus rhythm, which leads to discontinuation of OAC in majority of patients [8]. In the present study, maintained sinus rhythm after AF ablation was achieved in 67% of patients, and OAC was discontinued in 55.8% of patients at 5-year. The cumulative incidence of WRF at 1-, 3-, and 5-year was 3.5%, 8.5%, and 13.2%, respectively, which was much lower than that in a study by Yao, et al., with the cumulative incidence of WRF of 12–18% at 1-year and 22–26% at 2-year among AF patients treated with medical therapy including OAC [11]. We also assessed the impact of recurrent AF on renal function after AF ablation. Recurrent AF was an independent risk factor for WRF, which was in accordance with the sensitivity analysis of annual eGFR decline and previous reports by Park, et al and Kornej et al. [6, 7]. The possible mechanisms of this protective effect of sinus rhythm maintenance on renal function include elimination of AF followed by discontinuation of OAC, which may lead to reduced risks of renal hypoperfusion and micro embolism or bleeding. Thus, catheter ablation may break the vicious cycle between AF and CKD.

In the present study, we also evaluated the impact of WRF on long-term clinical outcomes after AF ablation. WRF, especially within 1 year post procedure, was independently associated with higher incidence of all the adverse clinical outcomes, such as all-cause and cardiovascular deaths, heart failure hospitalization, ischemic stroke, and major bleeding. In addition, we found that history of CHF as well as recurrent AF was an independent predictor of WRF and adverse clinical outcomes. Renal function is closely related to cardiac function, so-called cardio-renal syndrome [24, 25]. Also, AF is a well-known risk factor for exacerbation of CHF. This triangle association across AF, CHF, and WRF should be recognized in the management of AF.

The present study has several limitations. First, decline in eGFR was evaluated using a single SCr value during follow-up, which should have been influenced by body water at the time of measurement. Second, because all patients were Japanese in the present study, we used the JSN-CKDI equation for calculating eGFR, which is different from the equations outside Japan. Also, the mean age of patients of the present study was higher as compared with previous studies outside Japan, leading to lower baseline mean eGFR [5, 6, 26, 27]. Therefore, generalizing the results of the present study to populations outside Japan should be done with caution. Third, we did not have a control group of AF patients not undergoing catheter ablation. Fourth, repeat procedures for recurrent AF were performed in about 40% of patients during the follow-up period, which might have influenced the impact of maintaining sinus rhythm on WRF. Fifth, causal relationship between WRF and clinical outcomes was unclear because they were assessed using laboratory and clinical data during the same follow-up period. Finally, the multivariable analyses might have not adequately eliminated the influence of unmeasured confounders on determining the independent predictors of WRF and clinical outcomes following the ablation procedure. We cannot exclude the possibility that WRF was partly just a marker of sicker patients with socially and economically worse circumstances.

In conclusion, among patients undergoing catheter ablation for AF, arrhythmia recurrence was associated with WRF during follow-up, which was a strong predictor of subsequent adverse clinical outcomes.

## Supporting information

S1 Fig Prevalence of repeat catheter ablation procedures. (PPTX)

S2 Fig Cumulative incidences of >10%, >20%, and >30% declines in eGFR after AF ablation. (PPTX)

S3 Fig Cumulative incidence of OAC discontinuation comparing. A) patients with maintained sinus rhythm and those with recurrent AF; B) patients with and without WRF.
(PPTX)

S4 Fig Cumulative incidences of clinical outcomes after catheter ablation for AF comparing patients with WRF within 1 year post procedure relative to those without. A) all-cause death, B) cardiovascular death, C) heart failure hospitalization, D) ischemic stroke, and E) major bleeding. WRF = worsening renal function.
(PPTX)

S5 Fig Annual rate of eGFR change in patients with and without recurrent AF. (PPTX)

S6 Fig Cumulative incidences of clinical outcomes after catheter ablation for AF comparing patients with and without WRF. A) all-cause death, B) cardiovascular death, C) heart failure hospitalization, D) ischemic stroke, and E) major bleeding. WRF = worsening renal function.
(PPTX)

S1 Table Independent risk factors for worsening renal function after catheter ablation: A sensitivity analysis with all-cause death as a competing risk. (DOCX)

S2 Table Independent risk factors for worsening renal function after catheter ablation: A sensitivity analysis excluding AF patients with chronic kidney disease. (DOCX)

S3 Table Independent risk factors for heart failure hospitalization after catheter ablation for AF: A sensitivity analysis with all-cause death as a competing risk. (DOCX)

S4 Table Independent risk factors for worsening renal function after catheter ablation: A sensitivity analysis of annual rate of eGFR decline. (DOCX)

S5 Table Independent risk factors for all-cause death and heart failure hospitalization after catheter ablation for AF: A sensitivity analysis of annual rate of eGFR decline. (DOCX)

## Abbreviations

AAD: antiarrhythmic drug
AF: Atrial fibrillation
CHF: congestive heart failure
CKD: chronic kidney disease
DOAC: direct oral anticoagulant
eGFR: estimate glomerular filtration rate
OAC: oral anticoagulant
SCr: serum creatinine
WRF: worsening renal function
