# Supplementary figures and images for: Renal function and outcomes in atrial fibrillation patients after catheter ablation
Source: PLoS One. 2020 Nov 9;15(11):e0241449. doi: 10.1371/journal.pone.0241449 (PMC7652258; doi:10.1371/journal.pone.0241449)

## Slide 1
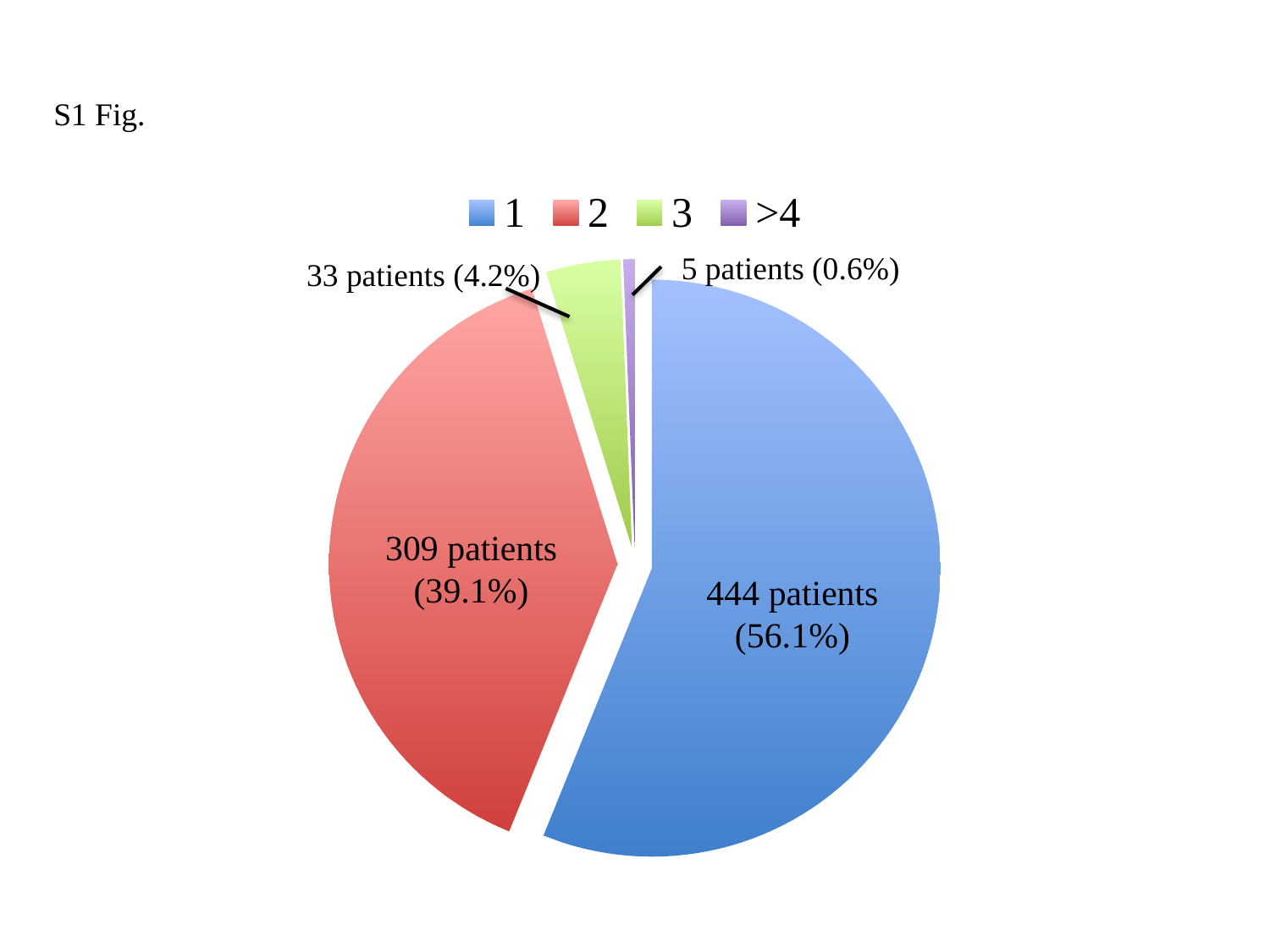

S1 Fig.
### Chart
| Category | 売上高 |
|---|---|
| 1 | 444.0 |
| 2 | 309.0 |
| 3 | 33.0 |
| >4 | 5.0 |5 patients (0.6%)
33 patients (4.2%)
309 patients
(39.1%)
444 patients
(56.1%)

Supplement: S1 Fig — (PPTX) [file pone.0241449.s001.pptx]

## Slide 1
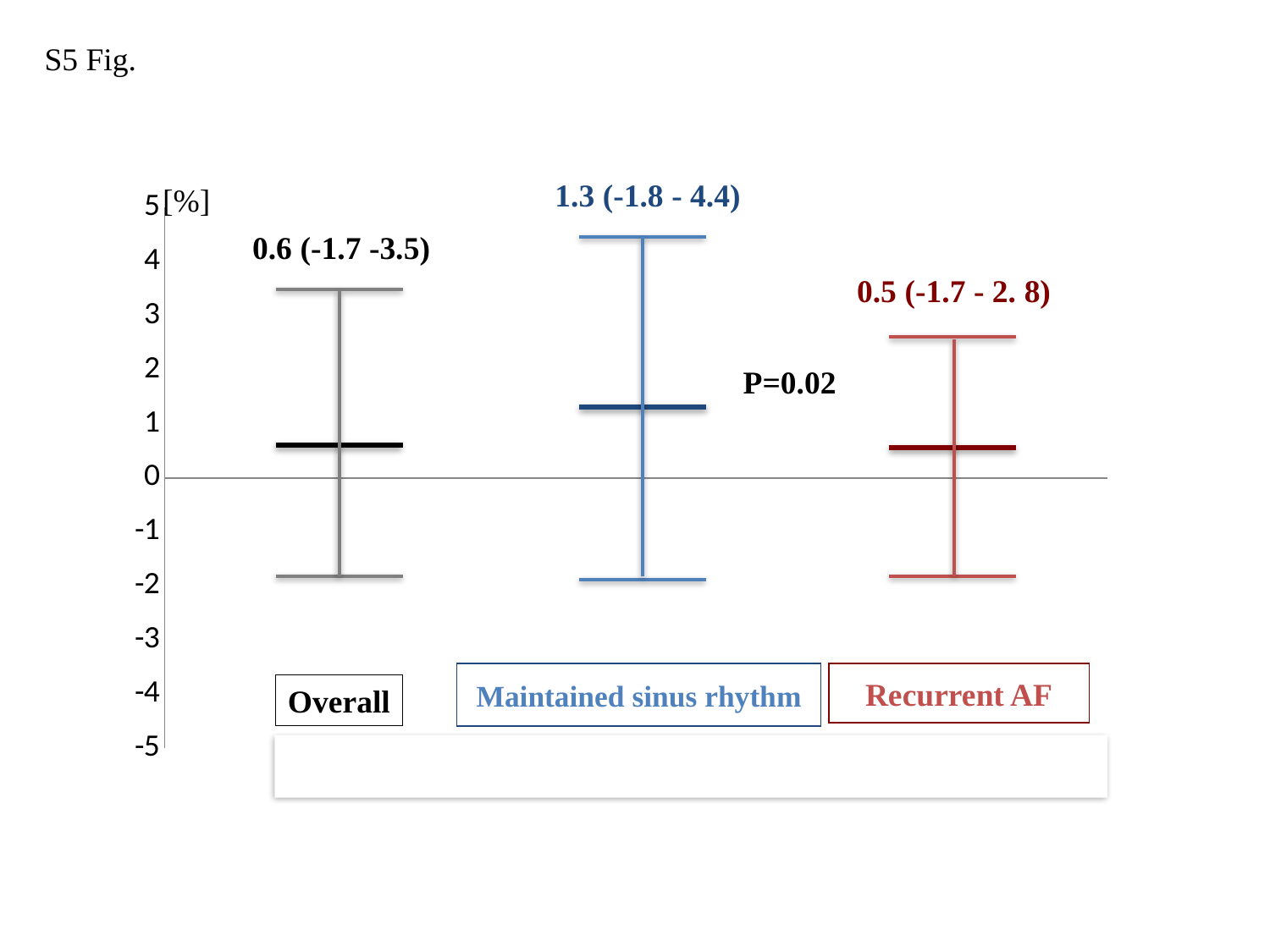

S5 Fig.
1.3 (-1.8 - 4.4)
[%]
### Chart
| Category | 系列 1 |
|---|---|
| Overall | 0.6 |
| SR | 1.3 |
| AF | 0.5 |0.6 (-1.7 -3.5)
0.5 (-1.7 - 2. 8)
P=0.02
Maintained sinus rhythm
Recurrent AF
Overall

Supplement: S5 Fig — (PPTX) [file pone.0241449.s005.pptx]
