# Supplementary material for: Renal function and outcomes in atrial fibrillation patients after catheter ablation
Source: PLoS One. 2020 Nov 9;15(11):e0241449. doi: 10.1371/journal.pone.0241449 (PMC7652258; doi:10.1371/journal.pone.0241449)
Supplement: S2 Fig — (PPTX) [file pone.0241449.s002.pptx]

## Slide 1
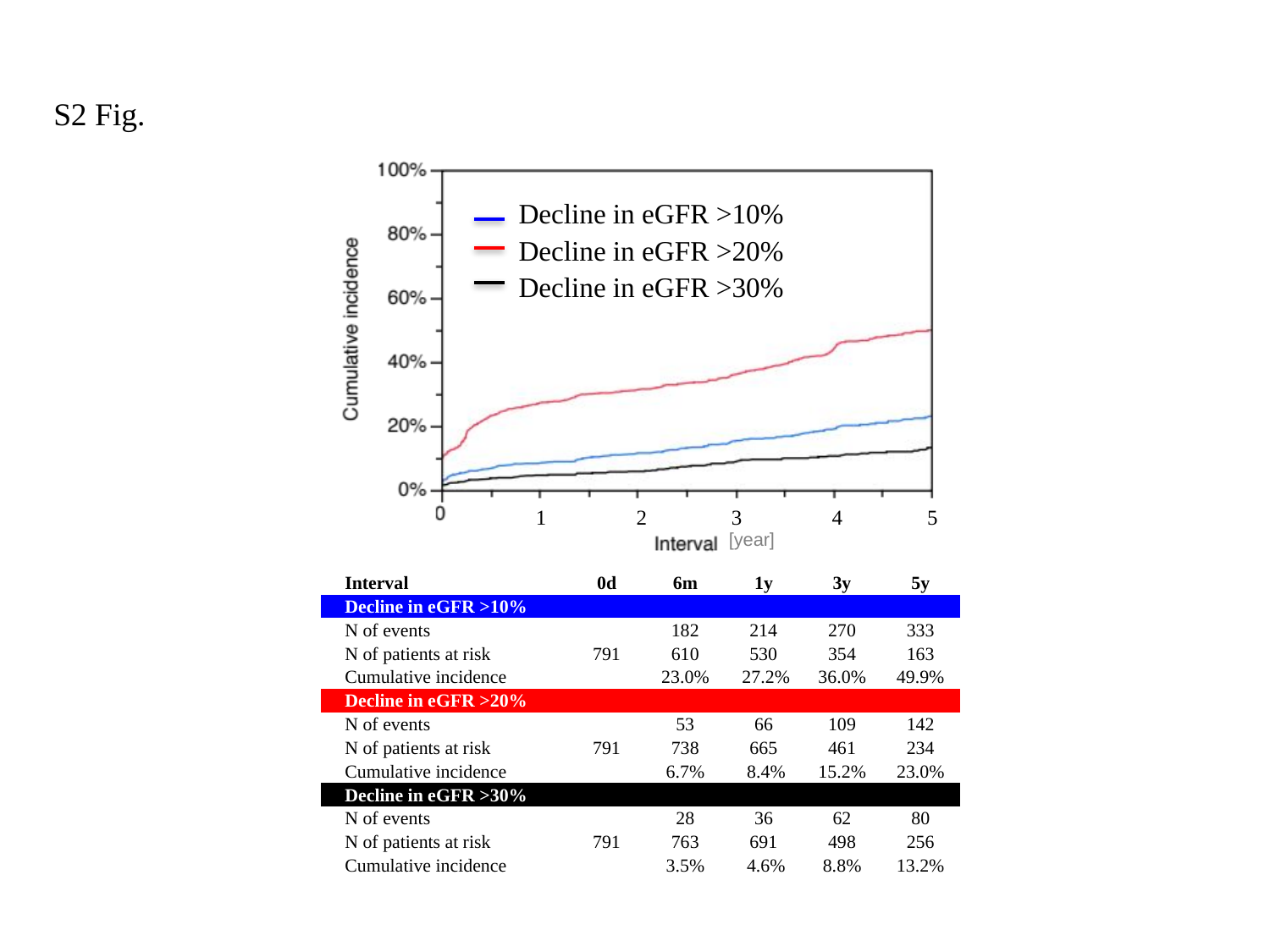

S2 Fig.
Decline in eGFR >10%
Decline in eGFR >20%
Decline in eGFR >30%
 1 2 3 4 5
[year]
| Interval | 0d | 6m | 1y | 3y | 5y |
| --- | --- | --- | --- | --- | --- |
| Decline in eGFR >10% | | | | | |
| N of events | | 182 | 214 | 270 | 333 |
| N of patients at risk | 791 | 610 | 530 | 354 | 163 |
| Cumulative incidence | | 23.0% | 27.2% | 36.0% | 49.9% |
| Decline in eGFR >20% | | | | | |
| N of events | | 53 | 66 | 109 | 142 |
| N of patients at risk | 791 | 738 | 665 | 461 | 234 |
| Cumulative incidence | | 6.7% | 8.4% | 15.2% | 23.0% |
| Decline in eGFR >30% | | | | | |
| N of events | | 28 | 36 | 62 | 80 |
| N of patients at risk | 791 | 763 | 691 | 498 | 256 |
| Cumulative incidence | | 3.5% | 4.6% | 8.8% | 13.2% |
