# Supplementary material for: Renal function and outcomes in atrial fibrillation patients after catheter ablation
Source: PLoS One. 2020 Nov 9;15(11):e0241449. doi: 10.1371/journal.pone.0241449 (PMC7652258; doi:10.1371/journal.pone.0241449)
Supplement: S3 Fig — A) patients with maintained sinus rhythm and those with recurrent AF; B) patients with and without WRF. (PPTX) [file pone.0241449.s003.pptx]

## Slide 1
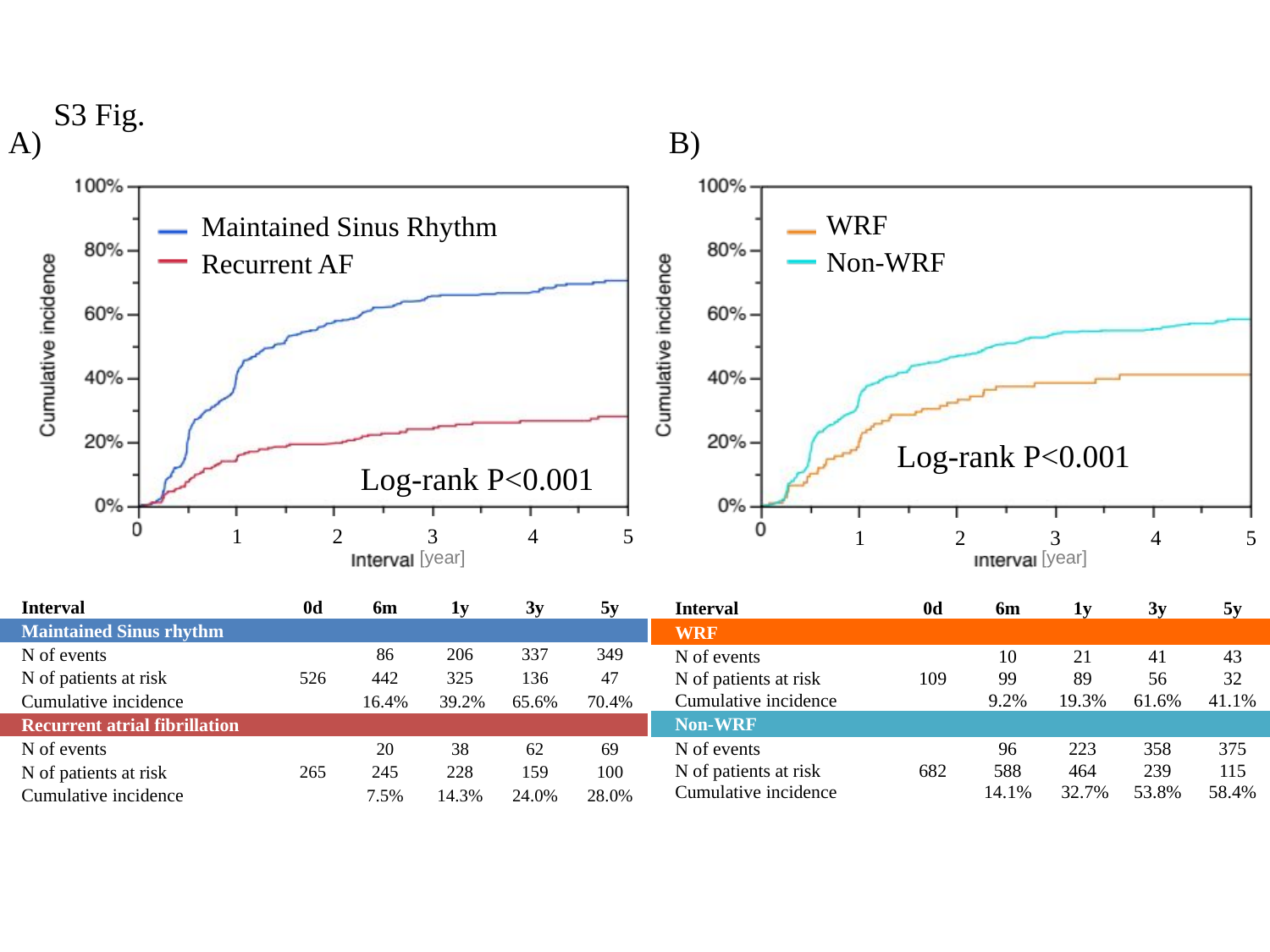

S3 Fig.
A)
B)
WRF
Non-WRF
Maintained Sinus Rhythm
Recurrent AF
Log-rank P<0.001
Log-rank P<0.001
 1 2 3 4 5
 1 2 3 4 5
[year]
[year]
| Interval | 0d | 6m | 1y | 3y | 5y |
| --- | --- | --- | --- | --- | --- |
| Maintained Sinus rhythm | | | | | |
| N of events | | 86 | 206 | 337 | 349 |
| N of patients at risk | 526 | 442 | 325 | 136 | 47 |
| Cumulative incidence | | 16.4% | 39.2% | 65.6% | 70.4% |
| Recurrent atrial fibrillation | | | | | |
| N of events | | 20 | 38 | 62 | 69 |
| N of patients at risk | 265 | 245 | 228 | 159 | 100 |
| Cumulative incidence | | 7.5% | 14.3% | 24.0% | 28.0% |
| Interval | 0d | 6m | 1y | 3y | 5y |
| --- | --- | --- | --- | --- | --- |
| WRF | | | | | |
| N of events | | 10 | 21 | 41 | 43 |
| N of patients at risk | 109 | 99 | 89 | 56 | 32 |
| Cumulative incidence | | 9.2% | 19.3% | 61.6% | 41.1% |
| Non-WRF | | | | | |
| N of events | | 96 | 223 | 358 | 375 |
| N of patients at risk | 682 | 588 | 464 | 239 | 115 |
| Cumulative incidence | | 14.1% | 32.7% | 53.8% | 58.4% |
