# Supplementary material for: Renal function and outcomes in atrial fibrillation patients after catheter ablation
Source: PLoS One. 2020 Nov 9;15(11):e0241449. doi: 10.1371/journal.pone.0241449 (PMC7652258; doi:10.1371/journal.pone.0241449)
Supplement: S4 Fig — A) all-cause death, B) cardiovascular death, C) heart failure hospitalization, D) ischemic stroke, and E) major bleeding. WRF = worsening renal function. (PPTX) [file pone.0241449.s004.pptx]

## Slide 1
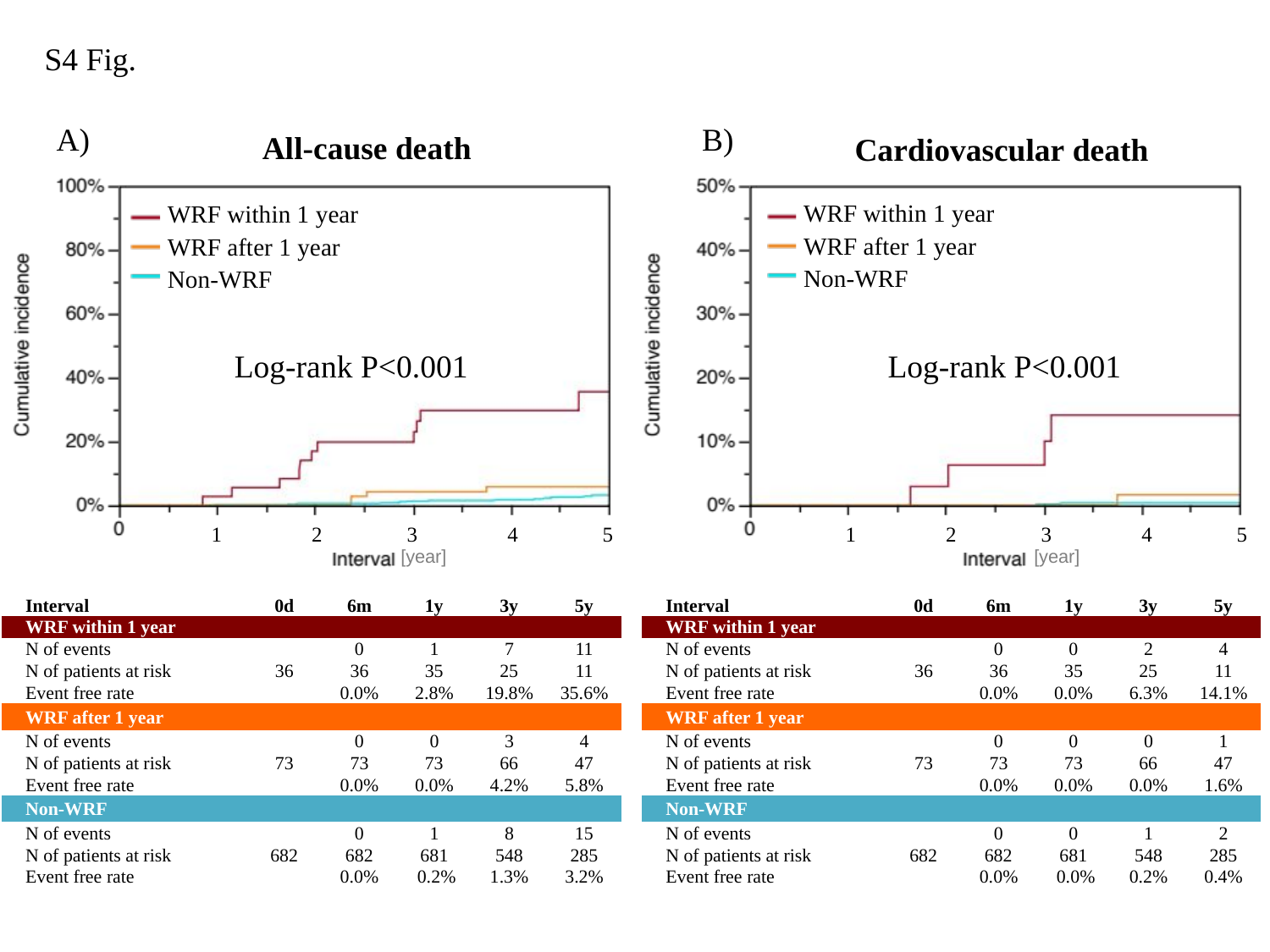

S4 Fig.
A)
B)
All-cause death
Cardiovascular death
WRF within 1 year
WRF after 1 year
Non-WRF
WRF within 1 year
WRF after 1 year
Non-WRF
Log-rank P<0.001
Log-rank P<0.001
 1 2 3 4 5
 1 2 3 4 5
[year]
[year]
| Interval | 0d | 6m | 1y | 3y | 5y |
| --- | --- | --- | --- | --- | --- |
| WRF within 1 year | | | | | |
| N of events | | 0 | 1 | 7 | 11 |
| N of patients at risk | 36 | 36 | 35 | 25 | 11 |
| Event free rate | | 0.0% | 2.8% | 19.8% | 35.6% |
| WRF after 1 year | | | | | |
| N of events | | 0 | 0 | 3 | 4 |
| N of patients at risk | 73 | 73 | 73 | 66 | 47 |
| Event free rate | | 0.0% | 0.0% | 4.2% | 5.8% |
| Non-WRF | | | | | |
| N of events | | 0 | 1 | 8 | 15 |
| N of patients at risk | 682 | 682 | 681 | 548 | 285 |
| Event free rate | | 0.0% | 0.2% | 1.3% | 3.2% |
| Interval | 0d | 6m | 1y | 3y | 5y |
| --- | --- | --- | --- | --- | --- |
| WRF within 1 year | | | | | |
| N of events | | 0 | 0 | 2 | 4 |
| N of patients at risk | 36 | 36 | 35 | 25 | 11 |
| Event free rate | | 0.0% | 0.0% | 6.3% | 14.1% |
| WRF after 1 year | | | | | |
| N of events | | 0 | 0 | 0 | 1 |
| N of patients at risk | 73 | 73 | 73 | 66 | 47 |
| Event free rate | | 0.0% | 0.0% | 0.0% | 1.6% |
| Non-WRF | | | | | |
| N of events | | 0 | 0 | 1 | 2 |
| N of patients at risk | 682 | 682 | 681 | 548 | 285 |
| Event free rate | | 0.0% | 0.0% | 0.2% | 0.4% |

## Slide 2
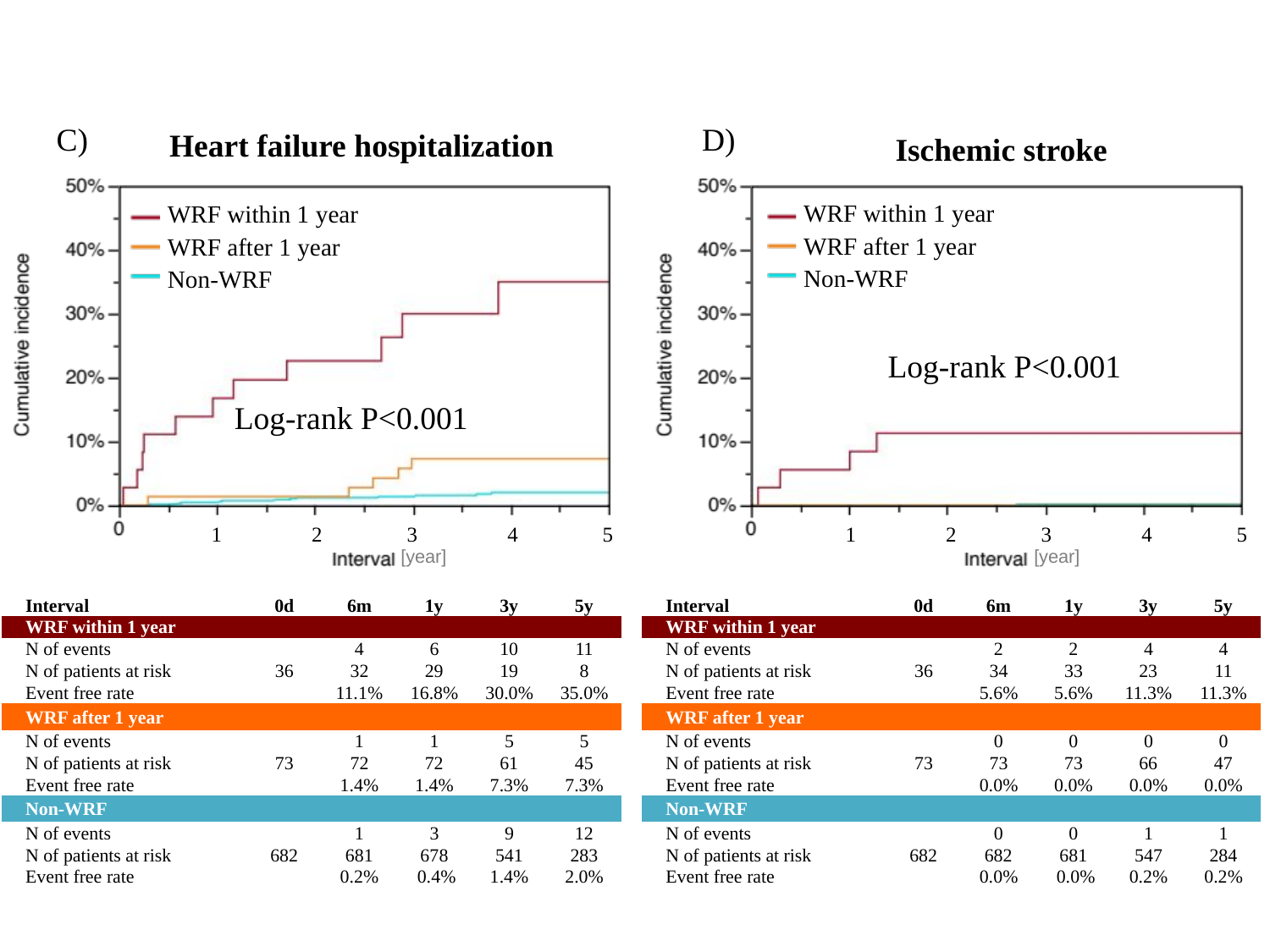

C)
D)
Heart failure hospitalization
Ischemic stroke
WRF within 1 year
WRF after 1 year
Non-WRF
WRF within 1 year
WRF after 1 year
Non-WRF
Log-rank P<0.001
Log-rank P<0.001
 1 2 3 4 5
 1 2 3 4 5
[year]
[year]
| Interval | 0d | 6m | 1y | 3y | 5y |
| --- | --- | --- | --- | --- | --- |
| WRF within 1 year | | | | | |
| N of events | | 4 | 6 | 10 | 11 |
| N of patients at risk | 36 | 32 | 29 | 19 | 8 |
| Event free rate | | 11.1% | 16.8% | 30.0% | 35.0% |
| WRF after 1 year | | | | | |
| N of events | | 1 | 1 | 5 | 5 |
| N of patients at risk | 73 | 72 | 72 | 61 | 45 |
| Event free rate | | 1.4% | 1.4% | 7.3% | 7.3% |
| Non-WRF | | | | | |
| N of events | | 1 | 3 | 9 | 12 |
| N of patients at risk | 682 | 681 | 678 | 541 | 283 |
| Event free rate | | 0.2% | 0.4% | 1.4% | 2.0% |
| Interval | 0d | 6m | 1y | 3y | 5y |
| --- | --- | --- | --- | --- | --- |
| WRF within 1 year | | | | | |
| N of events | | 2 | 2 | 4 | 4 |
| N of patients at risk | 36 | 34 | 33 | 23 | 11 |
| Event free rate | | 5.6% | 5.6% | 11.3% | 11.3% |
| WRF after 1 year | | | | | |
| N of events | | 0 | 0 | 0 | 0 |
| N of patients at risk | 73 | 73 | 73 | 66 | 47 |
| Event free rate | | 0.0% | 0.0% | 0.0% | 0.0% |
| Non-WRF | | | | | |
| N of events | | 0 | 0 | 1 | 1 |
| N of patients at risk | 682 | 682 | 681 | 547 | 284 |
| Event free rate | | 0.0% | 0.0% | 0.2% | 0.2% |

## Slide 3
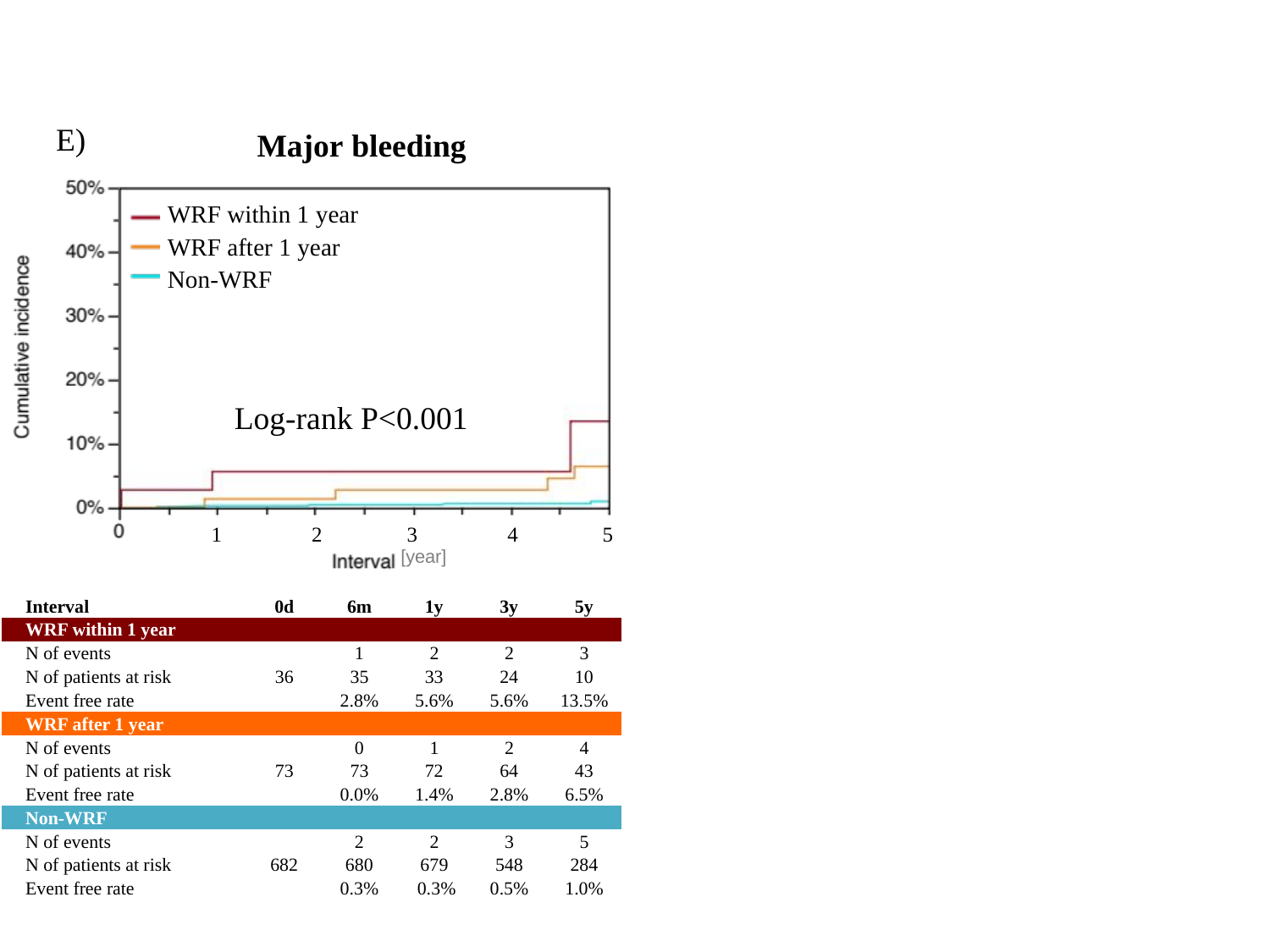

E)
Major bleeding
WRF within 1 year
WRF after 1 year
Non-WRF
Log-rank P<0.001
 1 2 3 4 5
[year]
| Interval | 0d | 6m | 1y | 3y | 5y |
| --- | --- | --- | --- | --- | --- |
| WRF within 1 year | | | | | |
| N of events | | 1 | 2 | 2 | 3 |
| N of patients at risk | 36 | 35 | 33 | 24 | 10 |
| Event free rate | | 2.8% | 5.6% | 5.6% | 13.5% |
| WRF after 1 year | | | | | |
| N of events | | 0 | 1 | 2 | 4 |
| N of patients at risk | 73 | 73 | 72 | 64 | 43 |
| Event free rate | | 0.0% | 1.4% | 2.8% | 6.5% |
| Non-WRF | | | | | |
| N of events | | 2 | 2 | 3 | 5 |
| N of patients at risk | 682 | 680 | 679 | 548 | 284 |
| Event free rate | | 0.3% | 0.3% | 0.5% | 1.0% |
