# Supplementary material for: Renal function and outcomes in atrial fibrillation patients after catheter ablation
Source: PLoS One. 2020 Nov 9;15(11):e0241449. doi: 10.1371/journal.pone.0241449 (PMC7652258; doi:10.1371/journal.pone.0241449)
Supplement: S6 Fig — A) all-cause death, B) cardiovascular death, C) heart failure hospitalization, D) ischemic stroke, and E) major bleeding. WRF = worsening renal function. (PPTX) [file pone.0241449.s006.pptx]

## Slide 1
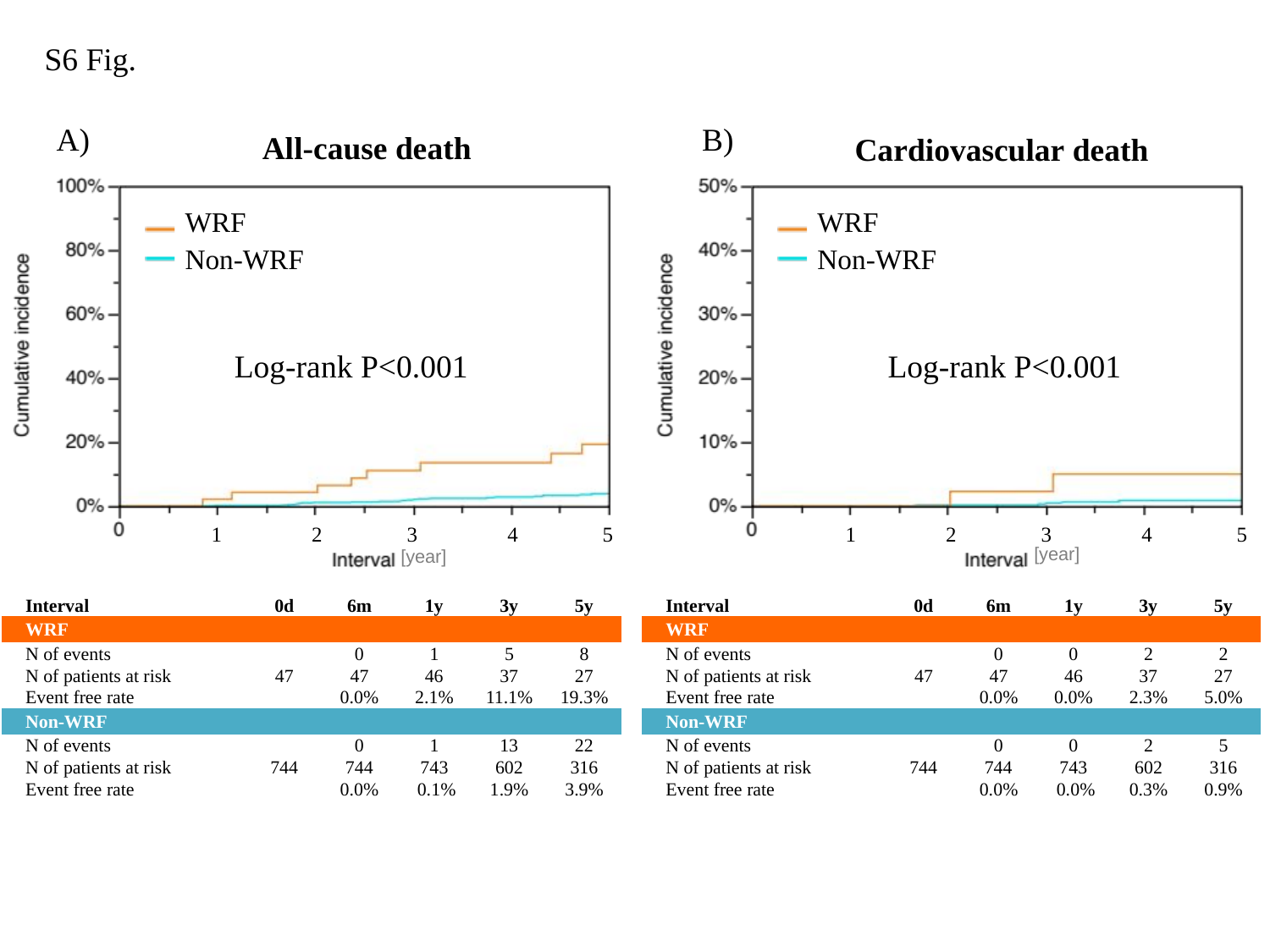

S6 Fig.
A)
B)
All-cause death
Cardiovascular death
WRF
Non-WRF
WRF
Non-WRF
Log-rank P<0.001
Log-rank P<0.001
 1 2 3 4 5
 1 2 3 4 5
[year]
[year]
| Interval | 0d | 6m | 1y | 3y | 5y |
| --- | --- | --- | --- | --- | --- |
| WRF | | | | | |
| N of events | | 0 | 1 | 5 | 8 |
| N of patients at risk | 47 | 47 | 46 | 37 | 27 |
| Event free rate | | 0.0% | 2.1% | 11.1% | 19.3% |
| Non-WRF | | | | | |
| N of events | | 0 | 1 | 13 | 22 |
| N of patients at risk | 744 | 744 | 743 | 602 | 316 |
| Event free rate | | 0.0% | 0.1% | 1.9% | 3.9% |
| Interval | 0d | 6m | 1y | 3y | 5y |
| --- | --- | --- | --- | --- | --- |
| WRF | | | | | |
| N of events | | 0 | 0 | 2 | 2 |
| N of patients at risk | 47 | 47 | 46 | 37 | 27 |
| Event free rate | | 0.0% | 0.0% | 2.3% | 5.0% |
| Non-WRF | | | | | |
| N of events | | 0 | 0 | 2 | 5 |
| N of patients at risk | 744 | 744 | 743 | 602 | 316 |
| Event free rate | | 0.0% | 0.0% | 0.3% | 0.9% |

## Slide 2
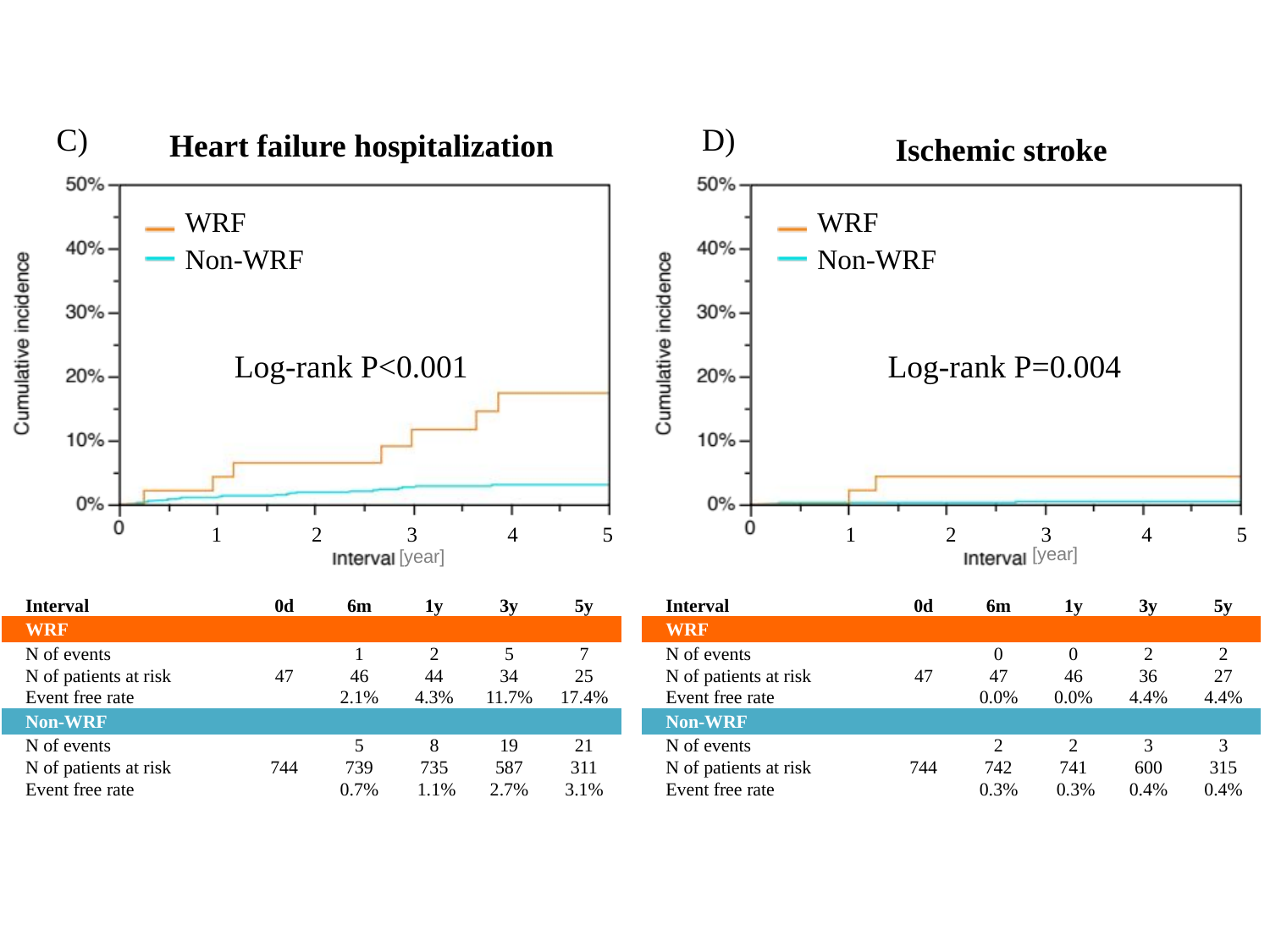

C)
D)
Heart failure hospitalization
Ischemic stroke
WRF
Non-WRF
WRF
Non-WRF
Log-rank P<0.001
Log-rank P=0.004
 1 2 3 4 5
 1 2 3 4 5
[year]
[year]
| Interval | 0d | 6m | 1y | 3y | 5y |
| --- | --- | --- | --- | --- | --- |
| WRF | | | | | |
| N of events | | 1 | 2 | 5 | 7 |
| N of patients at risk | 47 | 46 | 44 | 34 | 25 |
| Event free rate | | 2.1% | 4.3% | 11.7% | 17.4% |
| Non-WRF | | | | | |
| N of events | | 5 | 8 | 19 | 21 |
| N of patients at risk | 744 | 739 | 735 | 587 | 311 |
| Event free rate | | 0.7% | 1.1% | 2.7% | 3.1% |
| Interval | 0d | 6m | 1y | 3y | 5y |
| --- | --- | --- | --- | --- | --- |
| WRF | | | | | |
| N of events | | 0 | 0 | 2 | 2 |
| N of patients at risk | 47 | 47 | 46 | 36 | 27 |
| Event free rate | | 0.0% | 0.0% | 4.4% | 4.4% |
| Non-WRF | | | | | |
| N of events | | 2 | 2 | 3 | 3 |
| N of patients at risk | 744 | 742 | 741 | 600 | 315 |
| Event free rate | | 0.3% | 0.3% | 0.4% | 0.4% |

## Slide 3
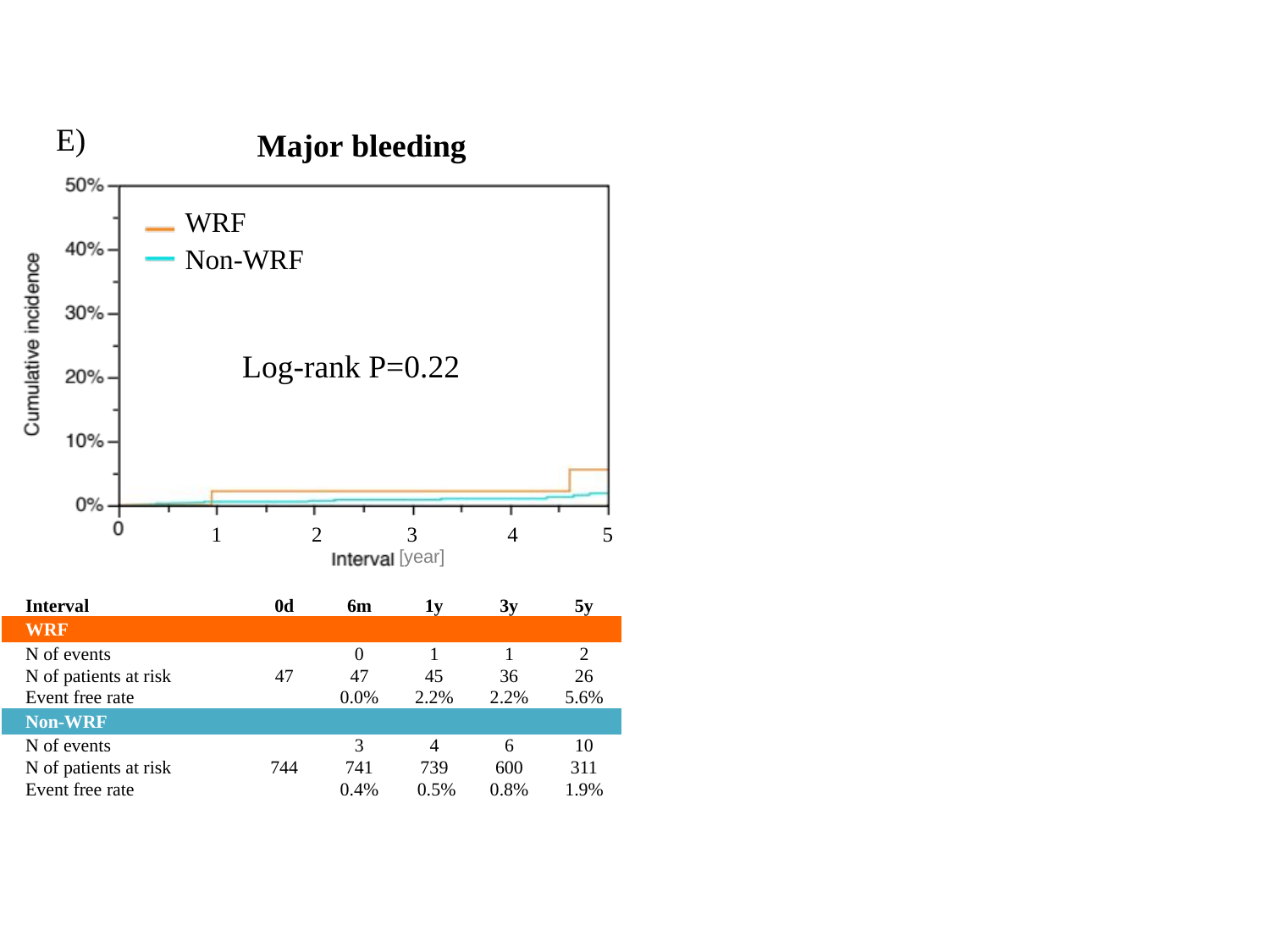

E)
Major bleeding
WRF
Non-WRF
Log-rank P=0.22
 1 2 3 4 5
[year]
| Interval | 0d | 6m | 1y | 3y | 5y |
| --- | --- | --- | --- | --- | --- |
| WRF | | | | | |
| N of events | | 0 | 1 | 1 | 2 |
| N of patients at risk | 47 | 47 | 45 | 36 | 26 |
| Event free rate | | 0.0% | 2.2% | 2.2% | 5.6% |
| Non-WRF | | | | | |
| N of events | | 3 | 4 | 6 | 10 |
| N of patients at risk | 744 | 741 | 739 | 600 | 311 |
| Event free rate | | 0.4% | 0.5% | 0.8% | 1.9% |
