# Supplementary material for: Renal function and outcomes in atrial fibrillation patients after catheter ablation
Source: PLoS One. 2020 Nov 9;15(11):e0241449. doi: 10.1371/journal.pone.0241449 (PMC7652258; doi:10.1371/journal.pone.0241449)
Supplement: S1 Table — (DOCX) [file pone.0241449.s007.docx]

**S1 Table. Independent risk factors for worsening renal function after catheter ablation: A sensitivity analysis with all-cause death as a competing risk.**

| **Variables** | **HR** | **95% CI** | **P value** |
| --- | --- | --- | --- |
| **Recurrent AF** | 1.89 | 1.27-2.82 | 0.002 |
| **Age >75 years old** | 1.08 | 0.62-1.87 | 0.78 |
| **Body mass index >25 kg/m^2^** | 0.88 | 0.57-1.36 | 0.56 |
| **Non-paroxysmal AF** | 1.24 | 0.81-1.91 | 0.32 |
| **Female** | 1.25 | 0.83-1.88 | 0.28 |
| **Hypertension** | 1.44 | 0.96-2.15 | 0.08 |
| **Diabetes** | 1.79 | 1.14-2.83 | 0.01 |
| **Congestive heart failure** | 2.87 | 1.82-4.53 | <0.001 |
| **Baseline CKD** | 1.46 | 0.97-2.19 | 0.073 |
| **Warfarin use** | 1.04 | 0.67-1.63 | 0.86 |

AF=atrial fibrillation; CI=confidence interval; CKD=chronic kidney disease; HR=hazard ratio.
