# Supplementary material for: Renal function and outcomes in atrial fibrillation patients after catheter ablation
Source: PLoS One. 2020 Nov 9;15(11):e0241449. doi: 10.1371/journal.pone.0241449 (PMC7652258; doi:10.1371/journal.pone.0241449)
Supplement: S2 Table — (DOCX) [file pone.0241449.s008.docx]

**S2 Table. Independent risk factors for worsening renal function after catheter ablation: A sensitivity analysis excluding AF patients with chronic kidney disease.**

| **Variables** | **HR** | **95% CI** | **P value** |
| --- | --- | --- | --- |
| **Recurrent AF** | 2.10 | 1.23-3.61 | 0.007 |
| **Age >75 years old** | 1.89 | 0.90-3.65 | 0.09 |
| **Body mass index >25 kg/m^2^** | 0.89 | 0.49-1.55 | 0.68 |
| **Non-paroxysmal AF** | 1.20 | 0.67-2.10 | 0.54 |
| **Female** | 1.13 | 0.65-1.92 | 0.65 |
| **Hypertension** | 1.13 | 0.67-1.91 | 0.65 |
| **Diabetes** | 2.29 | 1.28-3.95 | 0.006 |
| **Congestive heart failure** | 2.65 | 1.35-4.83 | 0.006 |
| **Warfarin use** | 1.01 | 0.57-1.82 | 0.98 |

AF=atrial fibrillation; CI=confidence interval; HR=hazard ratio.
