# Supplementary material for: Renal function and outcomes in atrial fibrillation patients after catheter ablation
Source: PLoS One. 2020 Nov 9;15(11):e0241449. doi: 10.1371/journal.pone.0241449 (PMC7652258; doi:10.1371/journal.pone.0241449)
Supplement: S3 Table — (DOCX) [file pone.0241449.s009.docx]

**S3 Table. Independent risk factors for heart failure hospitalization after catheter ablation for AF: A sensitivity analysis with all-cause death as a competing risk.**

| **Variables** | **Multivariable** | | |
| --- | --- | --- | --- |
|  | **HR** | **95% CI** | **P value** |
| **WRF** | 3.55 | 1.45-8.65 | 0.005 |
| **Recurrent AF** | 3.11 | 1.19-8.17 | 0.02 |
| **Age >75 years old** | 2.93 | 1.17-7.32 | 0.02 |
| **Non-paroxysmal AF** | 1.84 | 0.86-3.95 | 0.12 |
| **Diabetes** | 1.69 | 0.73-3.90 | 0.22 |
| **Congestive heart failure** | 6.28 | 2.89-13.7 | <0.001 |
| **Baseline CKD** | 2.03 | 0.94-4.40 | 0.073 |

AF=atrial fibrillation; CI=confidence interval; CKD=chronic kidney disease; HR=hazard ratio; WRF=worsening renal function.
