# Supplementary material for: Renal function and outcomes in atrial fibrillation patients after catheter ablation
Source: PLoS One. 2020 Nov 9;15(11):e0241449. doi: 10.1371/journal.pone.0241449 (PMC7652258; doi:10.1371/journal.pone.0241449)
Supplement: S4 Table — (DOCX) [file pone.0241449.s010.docx]

**S4 Table. Independent risk factors for worsening renal function after catheter ablation: A sensitivity analysis of annual rate of eGFR decline**

| **Variables** | **OR** | **95% CI** | **P value** |
| --- | --- | --- | --- |
| **Recurrent AF** | 2.19 | 1.16-4.16 | 0.02 |
| **Age >75 years old** | 0.62 | 0.21-1.54 | 0.33 |
| **Body mass index >25 kg/m^2^** | 0.84 | 0.42-1.60 | 0.60 |
| **Non-paroxysmal AF** | 0.65 | 0.31-1.28 | 0.21 |
| **Female** | 0.71 | 0.34-1.40 | 0.33 |
| **Hypertension** | 1.88 | 0.98-3.76 | 0.06 |
| **Diabetes** | 1.55 | 0.72-3.11 | 0.25 |
| **Congestive heart failure** | 1.56 | 0.60-3.58 | 0.34 |
| **Baseline CKD** | 0.51 | 0.23-1.04 | 0.06 |
| **Warfarin use** | 1.13 | 0.61-2.12 | 0.70 |

AF=atrial fibrillation; CI=confidence interval; CKD=chronic kidney disease; eGFR=estimated glomerular filtration rate; OR=odds ratio.
