# Supplementary material for: Renal function and outcomes in atrial fibrillation patients after catheter ablation
Source: PLoS One. 2020 Nov 9;15(11):e0241449. doi: 10.1371/journal.pone.0241449 (PMC7652258; doi:10.1371/journal.pone.0241449)
Supplement: S5 Table — (DOCX) [file pone.0241449.s011.docx]

**S5 Table. Independent risk factors for all-cause death and heart failure hospitalization after catheter ablation for AF: A sensitivity analysis of annual rate of eGFR decline**

1. **All-cause death**

| **Variables** | **Univariate** | | | **Multivariable** | | |
| --- | --- | --- | --- | --- | --- | --- |
|  | **HR** | **95% CI** | **P value** | **HR** | **95% CI** | **P value** |
| **WRF** | 5.69 | 2.50-11.8 | <0.001 | 5.50 | 2.38-11.6 | <0.001 |
| **Recurrent AF** | 2.26 | 1.08-4.53 | 0.03 | 1.55 | 0.76-3.20 | 0.23 |
| **Age >75 years old** | 1.58 | 0.59-3.58 | 0.34 |  |  |  |
| **Body mass index >25 kg/m^2^** | 0.81 | 0.36-1.69 | 0.59 |  |  |  |
| **Non-paroxysmal AF** | 1.05 | 0.48-2.14 | 0.90 |  |  |  |
| **Female** | 0.98 | 0.45-2.00 | 0.96 |  |  |  |
| **Hypertension** | 1.03 | 0.52-2.09 | 0.94 |  |  |  |
| **Diabetes** | 2.61 | 1.22-5.27 | 0.02 | 2.27 | 1.10-4.71 | 0.04 |
| **Congestive heart failure** | 4.38 | 1.98-9.01 | <0.001 | 2.91 | 1.34-6.35 | 0.01 |
| **Baseline CKD*** | 2.76 | 1.39-5.53 | 0.004 | 2.57 | 1.27-5.19 | 0.009 |
| **Warfarin use** | 2.21 | 1.00-5.59 | 0.05 |  |  |  |

**B) Heart failure hospitalization**

| **Variables** | **Univariate** | | | **Multivariable** | | |
| --- | --- | --- | --- | --- | --- | --- |
|  | **HR** | **95% CI** | **P value** | **HR** | **95% CI** | **P value** |
| **WRF** | 5.83 | 2.44-12.6 | <0.001 | 4.29 | 1.74-9.60 | 0.003 |
| **Recurrent AF** | 4.73 | 2.31-9.83 | <0.001 | 3.68 | 1.61-9.47 | 0.002 |
| **Age >75 years old** | 3.30 | 1.48-6.89 | 0.005 | 2.19 | 0.96-4.75 | 0.06 |
| **Body mass index >25 kg/m^2^** | 1.46 | 0.68-2.99 | 0.32 |  |  |  |
| **Non-paroxysmal AF** | 2.36 | 1.15-4.87 | 0.02 | 1.88 | 0.87-4.10 | 0.11 |
| **Female** | 0.96 | 0.42-2.04 | 0.92 |  |  |  |
| **Hypertension** | 1.27 | 0.62-2.77 | 0.52 |  |  |  |
| **Diabetes** | 2.32 | 1.01-4.09 | 0.048 | 1.90 | 0.81-4.15 | 0.13 |
| **Congestive heart failure** | 13.6 | 6.61-28.5 | <0.001 | 7.54 | 3.58-16.2 | <0.001 |
| **Baseline CKD** | 3.17 | 1.54-6.67 | 0.002 | 2.39 | 1.14-5.11 | 0.02 |
| **Warfarin use** | 1.47 | 0.70-3.31 | 0.32 |  |  |  |

**C) Heart failure hospitalization with all-cause death as a competing risk**

| **Variables** | **Multivariable** | | |
| --- | --- | --- | --- |
|  | **HR** | **95% CI** | **P value** |
| **WRF** | 3.94 | 1.33-11.7 | 0.01 |
| **Recurrent AF** | 3.83 | 1.58-9.28 | 0.003 |
| **Age >75 years old** | 2.31 | 0.96-5.53 | 0.06 |
| **Non-paroxysmal AF** | 1.92 | 0.81-4.52 | 0.14 |
| **Diabetes** | 1.94 | 0.80-4.74 | 0.15 |
| **Congestive heart failure** | 7.30 | 3.17-16.8 | <0.001 |
| **Baseline CKD** | 2.27 | 1.03-4.94 | 0.04 |

AF=atrial fibrillation; CI=confidence interval; CKD=chronic kidney disease; HR=hazard ratio; WRF=worsening renal function.
